# Learning Health System to rapidly improve the implementation of a school physical activity policy

**DOI:** 10.1186/s43058-024-00619-3

**Published:** 2024-07-31

**Authors:** Cassandra Lane, Nicole Nathan, John Wiggers, Alix Hall, Adam Shoesmith, Adrian Bauman, Daniel Groombridge, Rachel Sutherland, Luke Wolfenden

**Affiliations:** 1 School of Medicine and Public Health, The University of Newcastle, 1 University Drive Callaghan, Newcastle, NSW Australia; 2 Hunter New England Population Health, Hunter New England Area Health Service, Newcastle , NSW Australia; 3 National Centre of Implementation Science, The University of Newcastle, Newcastle, NSW Australia; 4 Hunter Medical Research Institute, New Lambton Heights, Newcastle, NSW Australia; 5 School of Public Health, University of Sydney, Sydney, NSW Australia

**Keywords:** Learning health systems, Implementation, Optimisation, Adaptations, School, Physical activity, Scale-up

## Abstract

**Background:**

Learning Health Systems (LHS) – characterised by cycles of evidence generation and application – are increasingly recognised for their potential to improve public health interventions and optimise health impacts; however there is little evidence of their application in the context of public health practice. Here, we describe how an Australian public health unit applied a LHS approach to successfully improve a model of support for implementation of a school-based physical activity policy.

**Methods:**

This body of work was undertaken in the context of a strong research-practice partnership. Core LHS capabilities included: i) partnerships and stakeholder engagement; ii) workforce development and learning health communities; iii) multi-disciplinary scientific expertise; iv) practice data collection and management system; v) evidence surveillance and synthesis; and vi) governance and organisational processes of decision making. Three cycles of data generation and application were used. Within each cycle, randomised controlled trials conducted in NSW primary schools were used to generate data on the support model’s effectiveness for improving schools’ implementation of a government physical activity policy, its delivery costs, and process measures such as adoption and acceptability. Each type of data were analysed independently, synthesised, and then presented to a multi-disciplinary team of researchers and practitioners, in consult with stakeholders, leading to collaborative decisions for incremental improvements to the support model.

**Results:**

*Cycle 1* tested the first version of the support model (composed of five implementation strategies targeting identified barriers of policy implementation) and showed the model’s feasibility and efficacy for improving schools’ policy implementation. Data-informed changes were made to enhance impact, including the addition of three implementation strategies to address outstanding barriers. *Cycle 2* (now, testing a package of eight implementation strategies) established the model’s effectiveness and cost-effectiveness for improving school's policy implementation. Data-informed changes were made to reduce delivery costs, specifically adapting the costliest strategies to reduce in-person contact from external support personnel. *Cycle 3* showed that the adaptations minimised the relative cost of delivery without adversely impacting on the effect.

**Conclusions:**

Through this process, we identified an effective, cost-effective, acceptable and scalable policy implementation support model for service delivery. This provides important information to inform or support LHS approaches for other agencies seeking to optimise the health impact of evidence-based interventions.

Contributions to the literature

- Demonstrates the application of a Learning Health System (LHS) approach in public health to improve policy implementation for preventive health services. This practical example sets an important precedent, as there are few real-world examples of LHS in practice, despite their considerable potential.
- Highlights the importance of a strong research-practice partnership in executing cycles of evidence generation and application; underlining the significance of collaborative partnerships in the field of implementation science, particularly within the context of preventive health.
- Showcases the success of randomised and controlled trials as an experimental method to generate robust data within each LHS cycle. This highlights the value of iterative trials in driving evidence-based decision-making and continuous improvement, enriching the methodology used in implementation science research.

## Introduction

Within healthcare more broadly, the World Health Organization (WHO) and Institute of Medicine have recommended the transformation of healthcare and other organisations into ‘Learning Health Systems’ (LHS) whereby research methods are applied to service, patient and/or population data to address and continually improve the impact of health services [1, 2]. LHS draw on approaches from a range of disciplines (i.e., implementation science, precision medicine, systems engineering) and utilise ongoing experimentation to efficiently build knowledge for health system improvement. It does this by undertaking research with and within health and community agencies, as part of their ‘usual business’, leveraging their expertise and resources to generate the knowledge that they use to continually improve services to address their priority issues.

Specifically, LHS are defined as systems in which "routine health practice data, from service delivery and patient care, can lead to iterative cycles of knowledge generation and healthcare improvement" [3]. They require a strong, collaborative partnership between research, healthcare organisations, and stakeholders [3, 4]. A distinguishing feature of LHS is the use of iterative, incremental, and data-driven processes of quality improvement, often as part of continual improvement cycles [5]. LHS frameworks have identified a number of key ‘pillars’ which represent enabling infrastructure to support LHS activities, specifically: scientific, social, technological, policy, and ethical [6]. While there is no singular model of a LHS, characteristics frequently cited by LHS frameworks as critical to their operation include stakeholder engagement, a skilled and agile workforce, systems of data collection and analysis (i.e., evidence generation), and data synthesis to inform improvement decisions [7–10].

The application of a LHS approach has also been identified as potentially transformative for public health policy and practice improvement [11]. Indeed, a group representing public health prevention agencies across Australian States and Territories has explicitly called for LHS approaches to generate the knowledge required to increase the uptake of effective preventive health interventions [12]. Health services that operate as a LHS, however, are uncommon [3, 4]. For example, a 2021 systematic review of evidence from high income countries [3] identified 23 LHS environments from 43 publications describing the benefits of LHS, none of which targeted the improvement of preventive health services or physical activity, nor sought to improve implementation in schools. The review concluded that while LHS can be a successful model, "there is limited evidence of effective systems level approaches and processes to deliver on these opportunities" [3]. The findings underscore the considerable challenges in applying LHS system approaches in the context of public health practice [13].

Given the paucity of examples of LHS for prevention generally, and community-based prevention specifically, we describe and reflect on a program of work undertaken by an Australian public health unit, Hunter New England Population Health (HNEPH), where a LHS approach has been applied. We illustrate this work using a case study of the iterative improvement of the implementation and impact of a school-based support model for physical activity policy implementation.

### Hunter New England population health

HNEPH is a government-funded regional health service in New South Wales (NSW), Australia. It is responsible for providing population-level prevention services for communicable and non-communicable disease across various settings, including schools [14]. HNEPH serves approximately 840,000 residents across the large (132,845 km^2^) and geographically diverse region of Hunter New England in NSW, Australia.

Since its establishment in 2005, HNEPH has operated as an embedded research-practice partnership, where researchers (typically employed by the University of Newcastle within the School of Medicine and Public Health) and HNEPH collaboratively develop, evaluate, and implement improved preventive health services [14]. HNEPH has invested in a range of strategies to support this partnership through collaborative innovation and research (See Table 1). These align with those commonly applied to enhance healthcare organisations engagement in research [15].

It has also invested in developing key organisational capabilities or ‘pillars’ suggested by LHS frameworks for efficient evidence generation and use by health services [16–18]. These include: i) partnerships and stakeholder engagement; ii) workforce development and learning health communities; iii) multi-disciplinary scientific expertise; iv) practice data collection and management system; v) evidence surveillance and synthesis; and vi) governance and organisational processes of decision making. This has enabled HNEPH to operationalise a LHS approach to optimise health prevention initiatives through data-driven rapid cycle testing of strategies to implement prevention programs in various community and clinical settings. This approach provides significant benefits for achieving service delivery objectives and accelerating research translation. It leverages both academic and health service expertise and resources, and builds the capacity of both entities [14].

**Table 1:** Research engagement strategies used by HNEPH

RESEARCH ENGAGEMENT STRATEGY	**APPLICATION BY HNEPH**
Including research-trained staff embedded/integrated within practice settings	• Academic staff allocated to research-practice roles are located in HNEPH sites and are integrated into service delivery teams and organisational governance positions. This includes staff in leadership roles with dual academic and health service appointments, who have expertise and responsibility for program design, implementation, evaluation, and improvement.
Provision of resources/infrastructure dedicated for research activity	• Academic appointments of HNEPH staff provide access to external research grants (funding) aligned to HNEPH innovation or improvement initiatives, as well as mentoring for research activity and ethics application support. • HNEPH funds and provides resources for core on-site research infrastructure (e.g., statistical support, data management and storage, and subscription to analytical software and bibliographic databases). • HNEPH provides in-kind cash investment in research activities aligned with service priorities.
Commitment, involvement and accountability of leaders and managers to support research.	• HNEPH has an explicit commitment to evidence-based practice (organisational mandate): **•** Staff in leadership roles endorse research in practice (see first row); **•** Research and evaluation are listed as core competencies and activities in staff position descriptions; and **•** Research activity is monitored and appraised as part of the annual performance review process.
Providing research- capacity building opportunities for practitioners (build practitioner competencies to undertake research)	• HNEPH has dedicated funds for staff professional development (e.g., attendance at workshops, scientific meetings, and conferences). • Training is delivered ‘in-house’ on a variety of research and practice skills (e.g., statistics, evidence synthesis, research co-production methods, and implementation science). • Membership is offered to jurisdiction-wide research and evaluation capacity building networks. • Staff are supported in undertaking post-graduate studies. • Staff have access to additional professional development opportunities through university appointments.
Networks and communication	• HNEPH engages in or hosts a range of academic and practice networks. • Joint supervision models are used for post-graduate students between HNEPH or University staff and other partner organisations. • Staff are represented on a range of academic, policy, and practice committees.
Partnership/collaborations between practice and academic institutions	• HNEPH uses material and in-kind resources to achieve shared goals with academic and other practice partners **•** Partnerships on research projects include: **•** Shared governance and leadership; and **•** Processes and agreements to support shared decision making.

### School-based physical activity program

We demonstrate the HNEPH LHS approach to service improvement through a case study on developing, evaluating, and implementing a strategy to enhance schools' adherence to state physical activity policies. In 2015, the NSW government released a Sport and Physical Activity Policy (hereafter, ‘the Policy’) requiring teachers to schedule 150 minutes of planned weekly physical activity for students in kindergarten to Grade 10 [19]. This could include time spent in physical education (PE), sport or other structured activity inclusive of all children such as evidence-based energisers (short bouts of 3-5 minutes of classroom physical activity breaks) or integrated lessons (physical activity integrated into other key learning areas, e.g., literacy or numeracy lessons) [8].

HNEPH has worked with schools to implement policies and practices promoting healthy eating and physical activity for over a decade. This has been supported by funding from the NSW Ministry of Health as part of large state-wide obesity prevention initiatives [20]. As part of their preventive service delivery, HNEPH identified the need to support schools to implement the Sport and Physical Activity Policy [19]. This need arose from evidence of enduring low levels of implementation within schools, derived from various sources including the published literature (e.g., an Auditor-General report [21]), feedback from key stakeholders, and local observations.

To address this, HNEPH embarked on a program of work to support schools in implementing the Policy. The goal was to develop an implementation support model consisting of multiple evidence-based implementation strategies that could be executed by HNEPH staff, which would be effective, acceptable, and capable of supporting all schools in the Hunter New England region given resource constraints. Specifically, HNEPH sought a model of support capable of improving the scheduling of weekly physical activity in a way that was acceptable to schools (as reported by principals) and that deployable to all approximately 400 schools in the Hunter New England region at the lowest possible cost to HNEPH.

## Methods

### Design

HNEPH applied a LHS approach to optimise the support model through a system of rapid, data-driven improvement ‘cycles’. Versions of the support model were developed, executed, evaluated, and adapted for improvement [5]. In total, three complete cycles were undertaken from 2017-2019 as part of this study. Broadly, these improvement cycles followed LHS processes comprised of three phases:

1. Knowledge to practice – Tacit knowledge, local data, prior evaluations, and evidence from the broader literature are used to develop and apply strategies to improve practice (policy implementation).
2. Practice to data – Data is collected to assess the extent to which such strategies have improved practice (implementation).
3. Data to knowledge – Data is analysed to generate new knowledge and identify opportunities for further practice improvement (policy implementation).

### LHS infrastructure, processes, and capabilities (pillars)

Each cycle of improvement utilised HNEPH infrastructure, core processes, and capabilities. These are aligned with other ‘pillars’ or LHS assets of LHS frameworks, particularly those with an implementation focus [16–18] including:[1] Partnerships and stakeholder engagement; [2] Workforce development and learning health communities; [3] Multi-disciplinary scientific expertise; 4] Practice data collection and management system; [5] Evidence surveillance and synthesis; [6] Governance and organisational processes of decision making. We describe the use of these pillars below, in the context of improving schools’ implementation of the Policy.

#### Partnerships and stakeholder engagement

The primary vehicle for stakeholder engagement was the establishment of an Advisory Group. This group provided insight and guided decision making regarding key aspects of implementation strategy development, execution, evaluation, and refinement across each cycle. The main target of the support model was the implementation of the Policy by schools and school staff, therefore, stakeholders from the education and health sector were identified at both the policy and local levels. The core group of 16 members was maintained throughout the project, consisting of, for the education-sector: policy-level representatives of the NSW Department of Education (as the Policy owner) and Catholic Schools Office, as well as local school principals and teachers in the Hunter New England region.; and, for the health sector: policy-level representatives through the NSW Office of Preventive Health, and local Hunter New England Local Health District personnel responsible for supporting the implementation of health initiatives in schools. The Group also included Aboriginal representation, aligning with the cultural governance model of HNEPH [22]. Aboriginal staff and stakeholders were engaged in decision making at all levels, providing insight and leadership to enhance the delivery of culturally appropriate services for Aboriginal communities. Additionally the group included academic experts in implementation science, physical activity, and health and education research. Membership turnover was addressed by replacing departing members with representatives from the same organisation to ensure consistent stakeholder representation.

Advisory Group members were engaged in the formative research used to design the support model; full details of this process are published elsewhere [23]. The strategies included in the support model addressed the identified barriers to the implementation of the physical activity Policy in schools and were selected based on member’s assessments of their potential effectiveness, feasibility, acceptability, costs, and relevance to the school context. Following the establishment of the support model (composed of a package of selected implementation strategies), routine quarterly online meetings were scheduled in advance and led by the project team (described following). These meetings served as a platform to provide stakeholders with updates and engage them in collaborative decision making processes to improve policy implementation strategies. This approach helped to ensure any changes remained effective, acceptable to end-users, and feasible to execute.

#### Workforce development and learning health communities

HNEPH invested in and developed the workforce and infrastructure to support iterative and ongoing improvement across cycles and to ensure inclusion of key informants during decision making processes. A project team was formed, comprising staff with multidisciplinary expertise to address specific areas of need to carry out the project consistent with LHS principles.

From within HNEPH, behavioural and implementation scientists were included to support the development, evaluation, and continual refinement of the support model. They reviewed the literature, engaged with the Advisory Group to develop logic models, and applied theoretical behaviour change frameworks to guide implementation strategy development and refinement thereafter. Health promotion practitioners with former employment experience as teachers in schools (hereafter ‘project officers’) were included to: provide insight into setting and content for the design and development of implementation strategies; execute the implementation strategies with schools; and provide input into evaluation methods. To support learning, practitioners and researchers engaged in the project were formally networked and met monthly within a single research-service team to reflect on implementation progress and share experiences and learnings.

#### Multi-disciplinary scientific expertise

External to HNEPH, independent researchers provided statistical and economic expertise informing evaluation methods and assessment of the clinical impact of the implementation strategy in relation to the economic constraints of delivery. Specifically, biostatistics input and trial methods advice from senior statisticians were outsourced from the Clinical Research Design, IT and Statistical Support (CReDITSS) Unit at the Hunter Medical Research Institute (HMRI). These statisticians provided expertise in causal inference, supporting the design of robust evaluations that could determine if and how the support model led to improvements in each cycle. Similarly, the services of health economists were also solicited from the HMRI Health Research Economics group, who supported resource use assessment and identified opportunities to improve efficiency.

#### Practice data collection and management systems

A system to collect data to evaluate and generate evidence of the effects of implementation strategies was crucial. We used sequential randomised controlled trials (RCTs) as the primary research design for the evaluation (‘study’ stage of the improvement cycle). Randomisation for each trial was performed by an independent statistician using a computer-based random number generator, with group allocation stratified by school characteristics such as geographic region and school size. Data from RCTs were used to generate new knowledge and inform improvements to the support model. The effect of the improvements made were then evaluated in subsequent RCTs. RCTs provided the most robust evidence of impact of the strategy and are recommended as the basis for healthcare decision making [10]. Improvements to implementation strategies made following each trial (‘act’ stage of the improvement cycle) were based on information collected in each trial and consideration of the broader research literature regarding the effectiveness of strategies to implement health interventions in schools [24].

HNEPH prioritised generating three types of evidence within each RCT: effectiveness in improving implementation of the Policy, process measures (e.g., acceptability and adoption), and delivery costs of the implementation strategy. Establishing effectiveness was considered essential to ensure the support model had a positive impact (i.e., the first trial) and then remained impactful following adaptations made to improve it (i.e., subsequent trials). The primary outcome used to assess the effect of the support model was minutes of scheduled weekly physical activity in all RCTs. In the absence of routinely collected data on teacher scheduling or activity (i.e., no existing data source for the outcome of interest), this was assessed using study specific data collection measures, specifically teacher logbooks [23]. This approach was decided by the Advisory Group and the multi-disciplinary project team based on use in other school-based studies [25–27], pragmatics as an acceptable and feasible approach within schools [28, 29], and offering reliability and consistency across trials [30]. Statisticians used linear mixed models to analyse differences between groups (intervention vs comparator) for the outcome measure. Within each RCT we also simultaneously collected data to provide evidence on the other two types (e.g., process measures and cost outcomes). Process evaluation measures [31, 32] were determined a priori by the project team, in consultation with the Advisory Group, based on identified areas of interest and stakeholder priorities. Across trials we consistently used quantitative methods (analysed via descriptive statistics) to measure acceptability, reach, and adoption. Acceptability was assessed from the perspective of school staff to determine the extent to which the support model and each of its implementation strategies were perceived positively and then remained acceptable. Reach and adoption of the strategy components were measured using project records, allowing us to assess the level of engagement (i.e., uptake of each component) by schools and/or school staff. We also drew on qualitative data from project officers and school staff (observations and insight of the implementers and end-users) to assess which components of support were perceived as viable and important for driving the effects of the support model. Qualitative data were obtained through anecdotal evidence from project officers' routine interactions with schools and through formal semi-structured interviews which were analysed thematically and following best practice guidelines for qualitative research and evaluation [33] and for achieving high information power [34].

Financial data were also collected to evaluate the absolute (total and cost-per-strategy) and relative costs (cost per increase in minute of time for physical activity scheduled) of delivering the support model over the 12-month intervention period. Cost data were collected through records of resource use maintained by the project team throughout the RCTs. This included labour (the time staff spent implementing strategies), travel and expenses incurred by staff, materials (printing and purchasing consumables), and teacher relief for attending workshops (i.e., reimbursement). This evidence was essential to justify the value of the investment by the health service providers and to identify opportunities to target implementation-strategy adaptations to reduce costs for successive delivery (described below).

#### Evidence surveillance and synthesis

In addition to the collection of data from local evaluations of each improvement strategy, evidence form the broader literature on the effects of strategies in improving the implementation of school-based physical activity program or polices was an important input. For this, we drew on a Cochrane review, including its updates, on strategies to improve the implementation of such interventions in schools[35], and other evidence reviews available at the time. This evidence ensured that decisions regarding improvements were guided by the best available evidence internationally, as well as data from our local evaluations.

#### Governance and organisational processes of decision making

As a government-funded regional health service in NSW responsible supporting policy implementation, ultimately, decisions regarding continued provision, or modifications to implementation support rested with HNEPH. However, these decisions were informed by both the findings of the research undertaken and the views and tacit knowledge of its stakeholders, and the Advisory group. As part of the evaluation step (‘study’ component) in the improvement cycles, each of the three types of evidence (effectiveness, process measures, and delivery costs) were independently analysed and interpreted by: statisticians for effectiveness data at the conclusion of each RCT; HNEPH practitioners and implementation scientists for process measures data; and HMRI health economists for delivery cost data. The findings of each analysis were then brought together and deliberative processes used to support their interpretation and guide opportunities to modify the support model to further enhance its impact and while reducing cost constraints. This involved appraising the relative effects of the strategy against the respective costs, incorporating the process measure indicators and qualitative insight of implementers. A written and visual portrayal of the merged evidence was developed and presented for final decisions, which were made including the Advisory Group.

## Results

**Fig. 1:**
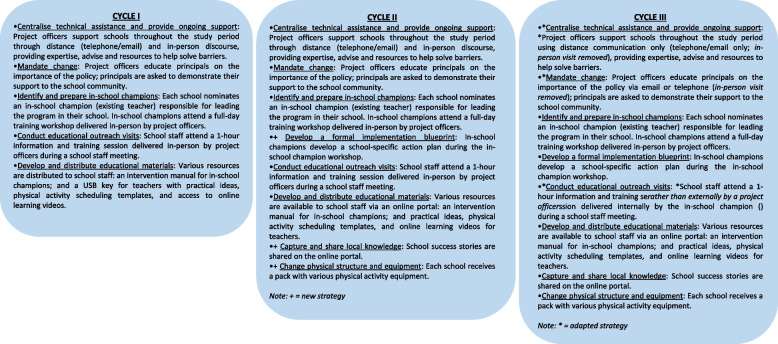
Implementation strategies used in the support model across cycles

The details for each trial, including study methods and findings, are published separately [23, 28–30, 36]. Figure 1 provides an overview of the support model and displays the changes to implementation strategies as the support model was optimised across trials.

### Cycle I

The first iteration of the support model comprised a package of five implementation strategies designed to address barriers that impeded teachers’ scheduling of weekly physical activity. These strategies included: ongoing assistance from project officers, principal support for the Policy following a meeting with project officers, in-school champions leading the program in their school after participating in a one-day training workshop, a one-hour training session for all teachers, and the provision of resources. A 2017 RCT with 12 Catholic primary schools allocated to receive the support model or a waitlist control group, provided empirical support for the efficacy of the support model in increasing schools’ implementation of the Policy [29]. Specifically, logbooks from 107 teachers showed a significant intervention effect: compared to controls, teachers from schools receiving the support model scheduled an additional 36.6 minutes of physical activity per week (95% CI, 2.7 to 70.5; p=.04). While the first iteration of the support model was effective, process data indicated that further enhancements could be made to help improve acceptability, reach, and potentially effectiveness. Additional barriers to implementation were identified (detailed below under Cycle II) that were perceived by the Advisory Group as not being adequately addressed by the current version of the support model.

### Cycle II

In the second iteration of the support model, several changes were made to enhance its impact whilst remaining feasible for delivery by HNEPH. Three strategies were added based on the evidence collected concerning the additional implementation barriers identified during the first RCT. One, school-specific action plans to implement the Policy and two, shared success stories from other schools were added to motivate teachers and address unforeseen challenges such as poor weather. Third, provision of a physical activity equipment pack was added to address the lack of readily accessible equipment for teachers. In addition to adding new strategies to the model, we enhanced the format and delivery of some existing strategies, including the development of an online portal to house educational materials and resources. This increased accessibility for school staff, enabled ongoing updates to resources, and provided an accountability and monitoring mechanism for schools.

A 2018 implementation-effectiveness RCT [37] was conducted with 61 primary schools allocated to receive the support model or a waitlist control group. The findings showed that the second iteration of the support model (now comprising eight implementation strategies) effectively increased teachers' minutes of scheduled physical activity [28]: teachers at schools receiving the support scheduled 44.2 minutes per week more physical activity than teachers at schools that did not receive the support (95% CI 32.8 to 55.7; p<0.001). An accompanying trial-based economic evaluation positioned the support model as a cost-effective approach for policy implementation [38], with an incremental cost-effectiveness ratio (ICER) of AUD $29 (95% uncertainty interval $17, $64) for every additional minute of weekly physical activity scheduled per school. Although the model was effective and cost-effective in supporting schools' implementation of the Policy, considerable scope remained to improve impact by enhancing capacity to reach a larger portion of the population ('scale-up') [39], while reducing relative cost and maintaining effectiveness.

### Cycle III

The effects achieved from the support model in Cycle II were considered acceptable by HNEPH and its stakeholders. However, there were some concerns about the absolute costs of the model when applied to all schools in the region. Based on process data and interpretation by the Advisory Group, all eight implementation strategies were considered important and acceptable. However, adaptations to the mode of delivery and format of several strategies were considered in an effort to improve reach and reduce cost - two constraints deemed essential by the Advisory Group to achieve a model of support that could be translated into real-world service delivery. Specifically, we sought to retain the impact of the support model but reduce its relative costs through mode of delivery adaptations. Thus, the third iteration of the support model reduced in-person contact from external support personnel by adapting the mode of delivery for three of the more costly implementation strategies from in-person to distance communication [38]. The ongoing assistance and the principal meeting were delivered by project officers via email/telephone rather than in-person, and the teacher training session was delivered by the in-school champion rather than an external project officer.

The effectiveness of the support model had been well established through the first two Cycles; therefore, a noninferiority randomised trial design was used (as per recommendations [40]) to test whether the third, adapted iteration was ‘as good as’ the proven effective support model (i.e., from Cycle II). A 2019 randomised controlled noninferiority trial was conducted with 48 primary schools (across two Local Health Districts) randomised to receive the adapted support model or the prior second iteration. Noninferiority was assessed using a linear mixed model analysed within a Bayesian framework. The findings showed that the adapted support model had a similar effect at increasing teacher's weekly minutes of scheduled physical activity, with a 96% probability of noninferiority for total physical activity and 99.6% probability of noninferiority for energisers [30]. Moreover, the adaptations minimised the relative cost of delivery by approximately AUD $373 per school: from $1057 for the second iteration of the support model to $684 for the third iteration [30]. In summary, the adapted support model (third iteration) was delivered in a more scalable and cost-effective format, without losing its meaningful effect on policy implementation.

## Discussion

This paper describes our application of the LHS approch to optimise a model of support for school’s physical activity policy implementation. This novel process provides a valuable contribution to implementation science and highlights the emergence of LHS as applied in the field of public health. The support model was incrementally improved using data from three cycles of evidence generation and application. Specifically, across these three cycles we enhanced the effect, feasibility and acceptability of the strategy while reducing the cost. These improvements were informed by three RCTs undertaken within the context of usual service delivery by a Health Promotion Unit and were achieved over a span of just three years (2017-2019). The findings demonstrate how a coordinated program of sequential trials, utilising harmonised methods and thoughtful stakeholder-engaged decision processes, can incrementally yet rapidly improve the impact of preventive services within real-world constraints, and generate new knowledge to advance implementation science.

These trials add substantively to the relatively small evidence base supporting the implementation of school-based physical activity policies [12, 35]. The application of LHS processes yielded an effective suite of implementation strategies comprising eight strategies (e.g., Principal support for the Policy, use of in-school champions, teacher training, provision of resources) adapted to be delivered more economically to schools. This provides agencies responsible for supporting the implementation of such polices with an effective and efficient means of doing so. A similar approach involving sequential trials has been employed to enhance the implementation of a health nutrition (canteen) policy in schools. In that case, a comprehensive suite of implementation strategies were found to be most effective in improving policy implementation, and changes to the modality of their delivery improved its cost effectiveness [41–43]. The findings are also consistent with systematic reviews of strategies to improve the implementation of prevention programs in schools more broadly, which have found relatively large improvements with multi-strategy approaches [35] and with other literature demonstrating similar impacts of interventions delivered using formats that require less (compared with more) ‘in-person’ support [44].

Underpinning the success of this project is the use of RCTs as the experimental design to empirically evaluate the support model within each cycle. RCTs provide robust evidence of effect [45] and are integrated into the routine practice of HNEPH. The findings suggests that use of such designs for public health improvement are possible. Another advantage of RCTs is the methodologically robust platform they provide to embed further assessments, thus maximising the information yielded from trial efforts [46]. Each trial was accompanied by evaluations of costs and process measures, revealing crucial information that informed the incremental improvements made to the support model across cycles. At the conclusion of Cycle III, the effective support model had been delivered to more than 200 schools (of which, 101 participated in trials evaluating impact) across three Local Health Districts in NSW, benefiting more than 70,000 students.

Although other experimental designs like adaptive trials and factorial experiments have value for optimising public health implementation strategies [5, 45], they are more complicated to enact and potentially disruptive to service provision. For example, the discrete implementation strategies of the support model being tested as part of the package to improve teachers’ delivery of physical activity were delivered at an organisational-level (schools) rather than individual-level (teacher). The tailoring of implementation strategies required in adaptive designs is not well suited for such interventions as it is typically not possible to expose organisational-level components to individual teachers without considerable risk of contamination. Sequential RCTs worked well in our context. That is, the sequential delivery, evaluation, and modification of implementation strategies over time represented a logistically feasible, albeit more protracted, means of optimisation compared to factorial experiments, where the simultaneous delivery and control of different combinations of strategies was considered prohibitively complex.

While the findings are encouraging, it is important to recognise that the work was undertaken within HNEPH where strong research-practice partnerships [14], conducive to application of LHS principles, and considerable research capacity are well established. Further, the infrastructure (e.g., workforce), partnerships (Department of Education and Health), and processes to support the execution of a program of improvement applying LHS principles have been developed and refined over many years. We share our experience here to help inform or support the development of such models of prevention service delivery for other agencies. We, and other prevention agencies in Australia, acknowledge that substantial investment and time would be required for a broader re-orientation of prevention services across the country [12].

### Reflection for future improvement

Following reflection on this body of work, we identified several areas for future improvement. LHS are not necessarily project specific, as described here, and further consideration is required for HNEPH to operate as an ingrained LHS, supporting optimised delivery of a range of health promotion activities. For example, throughout this project we included a range of stakeholders (exemplified by the diverse Advisory Group membership), ensuring that the perspectives of implementers (i.e., those necessary for implementation of the Policy) were incorporated in decision making processes. However, we did not engage with parents or students – members of the school community whose perspective would enhance understanding of the relevant needs and experiences of the school community. This is important information which could be used to inform and support health program service delivery for schools beyond teachers’ provision of physical activity. The engagement of a full range of consumers, such as through membership within the LHS learning health community (e.g., parent representatives in an Advisory Group), and including their perspective in the evidence generated (e.g., via qualitative interviews of parents and/or a focus group of students), represents an important opportunity for improvement. Further, in this project, school-level data (including the primary outcome data) were collected specifically for research purposes. A sustainable LHS for monitoring schools' implementation of the Policy will require establishing processes to support routine assessments of teachers’ scheduled minutes of physical activity.

Finally, although we sought to include a comprehensive range of evidence in our decision making, it is essential to include and prioritise evidence of the effects on equity to ensure health programs reach and are acceptable to priority populations. In our context, this might include schools in geographical rural/remote areas, low socioeconomic locations, and schools with a high proportion of Indigenous students. A focus on equity is imperative to address the gap in health outcomes experienced by historically underserved groups. LHS are well-positioned to achieve this [47], and a practical framework has been developed to support all stakeholders within a LHS to integrate and prioritise a focus on equity [47].

## Conclusions

In this paper we described our application of a LHS approach that resulted in an optimised policy implementation support model – one that is effective, cost-effective, acceptable, and scalable for health service delivery. The success of this body of work was facilitated by a strong research-practice partnership, stakeholder engagement, and the systematic application of LHS-driven improvement cycles. This work may serve as a model for other health agencies, providing valuable insight into the practical application of LHS principles to optimise the health impact of evidence-based interventions.

## Data Availability

The datasets used for the individual studies reported in the current study are available from the corresponding author on reasonable request.

## Abbreviations

LHS: Learning Health System
HNEPH: Hunter New England Population Health
RCT: Randomised controlled trial
NSW: New South Wales
CReDITSS: Clinical Research Design, IT and Statistical Support
HMRI: Hunter Medical Research Institute
